# Redondovirius-associated periodontitis in people with poor oral hygiene: a cross-sectional study

**DOI:** 10.3389/froh.2025.1572274

**Published:** 2025-05-23

**Authors:** Alireza Mohebbi, Zakiyeh Donyavi, Zoleikha Mamizadeh, Khadijeh Khanaliha, Nikoo Emtiazi, Seyed Jalal Kiani, Tahereh Donyavi, Alireza Shadab, Roghayeh Babaei, Farah Bokharaei-Salim

**Affiliations:** ^1^ Department of Virology, School of Medicine, Iran University of Medical Sciences, Tehran, Iran; ^2^ Vista Aria Rena Gene, Inc., Gorgan, Golestan, Iran; ^3^ Department of Endodontics, Clinical Research Development Unit, School of Dentistry, Alborz University of Medical Sciences, Kraj, Iran; ^4^ Department of Microbiology, Faculty of Advanced Science and Technology, Tehran Medical Science, Islamic Azad University, Tehran, Iran; ^5^ Research Center of Pediatric Infectious Diseases, Institute of Immunology and Infectious Diseases, Iran University of Medical Sciences, Tehran, Iran; ^6^ Department of Pathology, Iran University of Medical Sciences, Tehran, Iran; ^7^ Department of Medical Biotechnology, Faculty of Allied Medicine, Iran University of Medical Sciences, Tehran, Iran; ^8^ Department of Immunology, School of Medicine, Semnan University of Medical Sciences, Semnan, Iran; ^9^ Iran University of Medical Sciences, Deputy of Health, Tehran, Iran; ^10^ Department of Medical Nanotechnology, Faculty of Advanced Sciences and Technology, Tehran Medical Sciences, Islamic Azad University, Tehran, Iran

**Keywords:** redondoviridae, periodontitis, chronic periodontitis, oral hygiene, viral infection

## Abstract

**Background:**

This study aimed to investigate the prevalence of recently emerged members of the *Redondoviridae* family (ReDoVs) in individuals with periodontitis.

**Methods:**

This study involved real-world data from 230 participants, 115 with chronic periodontitis and an independent population consisting of 115 participants with oral hygiene habits selected from the refferals, and approved by the experts from October 2023 to May 2024. Demographic, health-related, and behavioral data were collected. Gingival samples were analyzed for ReDoVs using polymerase chain reaction (PCR), followed by sequencing and phylogenetic analysis.

**Results:**

ReDoVs were detected in 51.30% of the periodontitis group and 21.74% of the other groups with oral hygiene habits (*p* < 0.0001). ReDoV presence was associated with a lack of teeth-brushing habits (*p* < 0.05). Furthermore, flossing was negatively correlated with reduced ReDoV genomes (*r* = –0.20, *p* = 0.03).

**Conclusions:**

ReDoVs and periodontitis were associated with adults with poor dental hygiene. This supports a possible multifactorial and complex interaction between the presence of ReDoVs and periodontitis. Also, the findings of this study highlight that poor oral hygiene increases the likelihood of ReDoV presence and suggests that factors such as flossing are predictors of ReDoV infections.

## 1 Introduction

Redondoviruses (ReDoVs), which are part of the larger group of eukaryotic circular rep-encoding single-stranded (CRESS) DNA viruses with small genomes and diverse host ranges, have received much attention due to their prevalence in human biological samples and potential link to human health conditions. These viruses are distinguished by their tiny circular, single-stranded DNA genomes, roughly 3,000 nucleotides long. Studies have emphasized the broad distribution and possible adverse interactions of ReDoVs in humans, notably in respiratory and oral cavities (1, 2).

Recent studies show that ReDoVs are widely distributed in human populations, with varied prevalence rates in the respiratory tract, oral cavity, lungs, nasopharynx, and gastrointestinal system. Abbas et al. observed a 3.8% prevalence of ReDoV in the oral cavity, which is of great importance given the oral cavity's significance as a gateway for numerous infections (2). The recent discovery of two distinct *Redondoviridae* species, Vientovirus and Brisavirus, indicates that these viruses may each interact with hosts in unique ways, complicating the picture of their spread and behavior (2). Mapping the spread of these viruses is crucial for understanding the viral niches and the dynamics of their interactions with humans. Reports by (3, 4) found these viruses not only in healthy individuals but also in people with illnesses, indicating that they exist as part of a virome and their involvement in human-related diseases remains poorly understood (3, 4).

ReDoVs' clinical consequences are not known. Critical observations include increased viral loads in people with worse clinical outcomes, raising whether these viruses indicate illness severity or contribute to disease pathogenesis. This uncertainty highlights the need for more focused studies to better understand their function in human health and disease processes (5). Although their presence is associated with periodontitis and other significant conditions have not yet been definitively established. Research into their function in inflammatory illnesses suggests that ReDoVs may affect disease processes, perhaps aggravating or altering immune responses in ways that might complicate patient outcomes in diseases such as COVID-19 and chronic respiratory problems (6, 7).

The oral cavity has a diverse microbiome composed of bacteria, fungi, and viruses that play critical roles in oral disease and health. Periodontal disease, a prominent worldwide health concern, is caused mostly by bacterial infections in tooth plaque. However, current research revealed a complicated interplay between diverse microbial species, including viruses, contributing to the etiology of periodontal diseases. Periodontitis, a common chronic disease characterized by gum inflammation and tooth loss, has been the primary focus of research on the complex interplay between microbial dysbiosis and immunological responses. ReDoVs have been discovered in the oral cavity, where their interactions with the microbial flora and the host immune system may affect the course and severity of periodontal disease. Studies have revealed that these viruses might enhance the inflammatory response in the gums, thus accelerating overall tissue damage and disease development (8, 9).

Despite growing knowledge of ReDoVs, there are gaps in the direct relationship between these viruses and periodontal disease and contributing risk factors. Most investigations have been correlational rather than definitive, highlighting the need for further targeted study into the processes by which ReDoVs impact periodontal disease (2). There are still gaps to cover to support ReDoVs association with human diseases further (10, 11). The current work fills this gap by applying molecular methods to measure ReDoVs prevalence and strain diversity in gum samples from patients with periodontal disease and a group of selected participants who had good oral hygiene habits, intending to shed light on ReDoVs involvement in the etiopathogenesis of periodontitis.

This study's assumptions include the anticipation of a significant association between ReDoV prevalence and periodontal disease severity (7). Since the possible association between ReDoVs and dental complications is already addressed (6, 12–14), it was hypothesized that ReDoVs would be more prevalent in individuals with chronic periodontitis, especially those with poor oral hygiene. Therefore, this cross-sectional study designed to assess the effect of oral hygiene measures on the occurrence of ReDoVs, for which a group of individuals with oral hygiene habits was chosen alongside a group of referrals with periodontitis.

## 2 Methods

### 2.1 Study design, sample size, and data collection

Building on our earlier finding that ReDoVs may be linked to dental issues in patients screened for COVID-19 (14), this cross-sectional study was designed to to explore this relationship further. To achieve this, virologists from Iran University of Medical Sciences (IUMS) collaborated with endodontics researchers at Alborz University of Medical Sciences in Karaj (ABZUMS). Accordingly, 230 referrals to the School of Dentistry from October 2023 to May 2024 were asked to participate in the study. The participants gave their informed consent and were orally informed of the study. In this regard, participants could leave the study at their request, and their information would not be included in the investigation. A group of randomly selected referrals referred for routine checks to the School of Dentistry, Alborz University of Medical Sciences, Karaj, Iran, with recognizable periodontal problems formed one group. A separate group of participants with clinically healthy gingiva (no signs of periodontitis, caries, or other oral diseases) was selected based on radiographic and clinical examinations. Each group was comprised of 115 participants, 230 in total.

Participant selection was not biased by lifestyle, hygiene habits, or underlying diseases. Data were collected through a structured questionnaire and clinical examinations. Participants in this study were assessed based on demographical [age, sex, occupation, marital state, and intravenous drug users (IVDUs)], hygiene habits or health-related variables (dental floss usage, teeth brushing, smoking, and periodontitis severity), and medical history of underlying diseases like systemic conditions. For participants who reported systemic conditions, no detailed information on disease control was collected.

### 2.2 Inclusion and exclusion criteria

According to the staging criteria for periodontitis (15), the periodontitis group comprised those with chronic periodontitis. The participants were aged 18–65 years. Individuals suffering from human immunodeficiency virus (HIV) infection or malignancies, and pregnant women were excluded due to their independent effects on immune function and periodontal health. Conditions with no direct oral relevance, were retained. In addition, individuals who reported a history of periodontal treatment, smoking (>15 cigarettes/day), or antibiotics treatment (during the previous 6 months) were also excluded. Clinical examination was performed for all participants, and the periodontal health was evaluated, including the pocket depth (PD), bleeding on probing (BOP), plaque index (PLI), gingival index (GI), and clinical attachment loss (CAL). Radiographic assessment (orthopantomogram) was performed for both groups to confirm alveolar bone status. Accordingly, the group with periodontitis were diagnosed based on clinical and radiographic findings in line with the 2018 AAP case definition of periodontitis. Specifically, patients in the periodontitis group had bleeding on probing, mean pocket depth ≥6 mm, clinical attachment loss ≥3 mm, and radiographic alveolar bone resorption, approximately corresponding to Stage III periodontitis under the 2018 classification. In contrast, individuals showing a healthy periodontium, characterized by the absence of bleeding on probing, no clinical attachment loss, and a mean pocket depth of ≤4 mm, were also included for comparison with the periodontitis group regarding ReDoVs infection.

### 2.3 Sample collection and genome extraction

A skilled dentist gently took the samples from the gum with a sterile brush. Samples were immediately transferred into a vial containing viral transport media (VTM) and frozen at a temperature of −80°C for DNA extraction. According to the manufactured protocol, the viral DNA was extracted from the samples retrieved from participants using the QIAamp® DNA Mini kit (Qiagen GmbH, Germany). The quality of the extracted DNA was determined by NanoDrop (Thermo Scientiﬁc, Wilmington, MA) as described before (16).

### 2.4 Detection of the ReDoV by polymerase chain reaction

The presence of the ReDoV(s) genome was examined by our sensitive in-house polymerase chain reaction (PCR) assay, as described before (14). Briefly, the assay was done in a 25 µl reaction containing 10 pmol of primers, 2.5 µl of 10× Ex *Taq* buffer (Mg2 + free), 1.0 U of Ex *Taq* DNA polymerase (TaKaRa Biotechnology, Dalian Co., Ltd., Shiga, Japan), 2.0 µl of MgCl2 (25 mM), 2.0 µl dNTPs Mixture (25 mM each), and 5 µl of extracted viral DNA as template. To ensure accuracy, each PCR run included both a positive control, DNA from a confirmed ReDoV-positive sample, and a negative control (no-template water). Strict contamination prevention measures were followed, including physical separation of pre- and post-PCR work areas and use of filtered pipette tips. No amplification was observed in any negative control, indicating that contamination did not occur.

The thermal cycles were comprised of an initial denaturation at 95°C for 4 min, 35 cycles at 95°C for 30 s, at 56°C for 30 s, and at 72°C for 35 s, followed by a final extension at 72°C for 5 min. The PCR products of the specimens, negative and positive controls, and size marker (100 bp) were electrophoresed on 1.8% Agarose gel, stained using gel red, and visualized by an ultraviolet transilluminator.

### 2.5 Sequencing and phylogenic analysis

According to the manufactured protocol, 10 positive samples were randomly selected and purified using the high-pure PCR product purification kit (Roche Diagnostic, Mannheim, Germany). The purified samples were sequenced bi-directionally by the chain termination method using the ABI 3730 XL sequencer.

The taxonomical data of ReDoVs are classified in different oral-, lung-, and human respiratory-associated, as well as unassigned ReDoVs' sequences (14). Here, we have updated the ReDoVs' genomes to see if any more entries were from the NCBI Taxonomy database. A multiple sequence alignment (MSA) was performed with MAFFT v7 (17, 18) using the retrieved data from NCBI's Taxonomy database, our previously published ReDoVs sequencing data from the referrals for COVID-19 screenings, and data from the present study. Accordingly, MSA was performed with the G-INS-1 strategy to establish an accurate guide tree as described before (19). The statistical significance of the phylogenetic tree was evaluated using the bootstrap method (1,000 replicates). A closely related *Anellovirus* genome was also used as the outgroup to root the tree. A Newick graph-theoretical trees file format was saved to reconstruct the radial phylogram using the Interactive Tree Of Life (iTOL; https://itol.embl.de/) online tool (20).

### 2.6 Statistical analysis

Statistical analysis was performed by MS-Excel XLSTAT v2019 software package, as reported before (21, 22). The normality of the quantitative variable (age) was tested using the Kolmogorov–Smirnov test. The statistical differences between categorical variables were assessed using the Contingency table, and the strength of the test was approved by Fisher's exact test. An OR was calculated to compare the odds of ReDoV positivity between participants with periodontitis and healthy participants. The Miettinen-Nurminen method was used to compute the odds ratio's 95% confidence interval (CI). A hypothesis test was conducted using a score *Z* test to assess whether the odds of ReDoV positivity differed significantly between the two groups. The null hypothesis (H0) stated that the odds ratio in the two populations was equal to 1 (*ψ* = 1), meaning there was no association between periodontitis and ReDoV positivity. The alternative hypothesis (H1) proposed that the odds ratio was not equal to 1 (*ψ* ≠ 1), indicating a potential association. The Z statistic was calculated, and the corresponding *p*-value was obtained. Furthermore, multivariate Pearson correlation coefficients were calculated separately for healthy and periodontitis groups with 95% CI. A *p*-value of less than 0.05 was considered statistically significant.

### 2.7 Ethical considerations

The present study was approved ethically by the Ethics Committee, School of Medicine, IUMS, Tehran, Iran, by the Second Declaration of Helsinki, using the ethical code of IR.IUMS.REC.1402.530.

## 3 Results

### 3.1 Report of the retrieved data

Based on the specialist's clinical examinations, 115 people were randomly selected and consented to participate in this study from referrals recognized with chronic periodontitis. A further group of 115 randomly selected participants was also made with no dental or gum complications. It was checked that all participants filled out the questionnaire (*see* Supplementary File 1) to evaluate the variables toward the parameters regarding lifestyle, hygiene habits, and underlying diseases. The mean age of the participants was 37.11 ± 13.48 years old (minimum of 10 years old and maximum of 71 years old). Also, the mean age of periodontitis and healthy groups was 41.05 ± 13.75 and 33.17 ± 13.48, respectively. The groups were not age-matched (test value = 4.43, *p*-value < 0.0001), but statistical comparisons have been made for the sake of the cross-sectional study. The demographical reports of the participants are demonstrated in Table 1.

**Table 1:** Demographical information of the total participants.

Variable\Statistic	Nbr. of observations	Categories	Frequency per category	Rel. frequency per category (%)
Sex	230	Female	137	59.57
		Male	93	40.43
Dental floss usage	230	No	124	53.91
		Sometimes	19	8.26
		Yes	87	37.83
Age category	230	1–10	3	1.30
		11–19	7	3.04
		20–29	66	28.70
		30–39	70	30.43
		40–49	40	17.39
		50–65	36	15.65
		>65	8	3.48
Occupation	230	Employed	214	93.04
		Retired	11	4.78
		Unemployed	5	2.17
Marital state	230	Married	130	56.52
		Single	97	42.17
		Widow	3	1.30
Teeth brushing	230	>Once	59	25.65
		None	40	17.39
		Once	131	56.96
Smoking history	230	No	199	86.52
		Yes	31	13.48
IVDU^£^ history	230	No	229	99.57
		Yes	1	0.43

**Figure 1:**
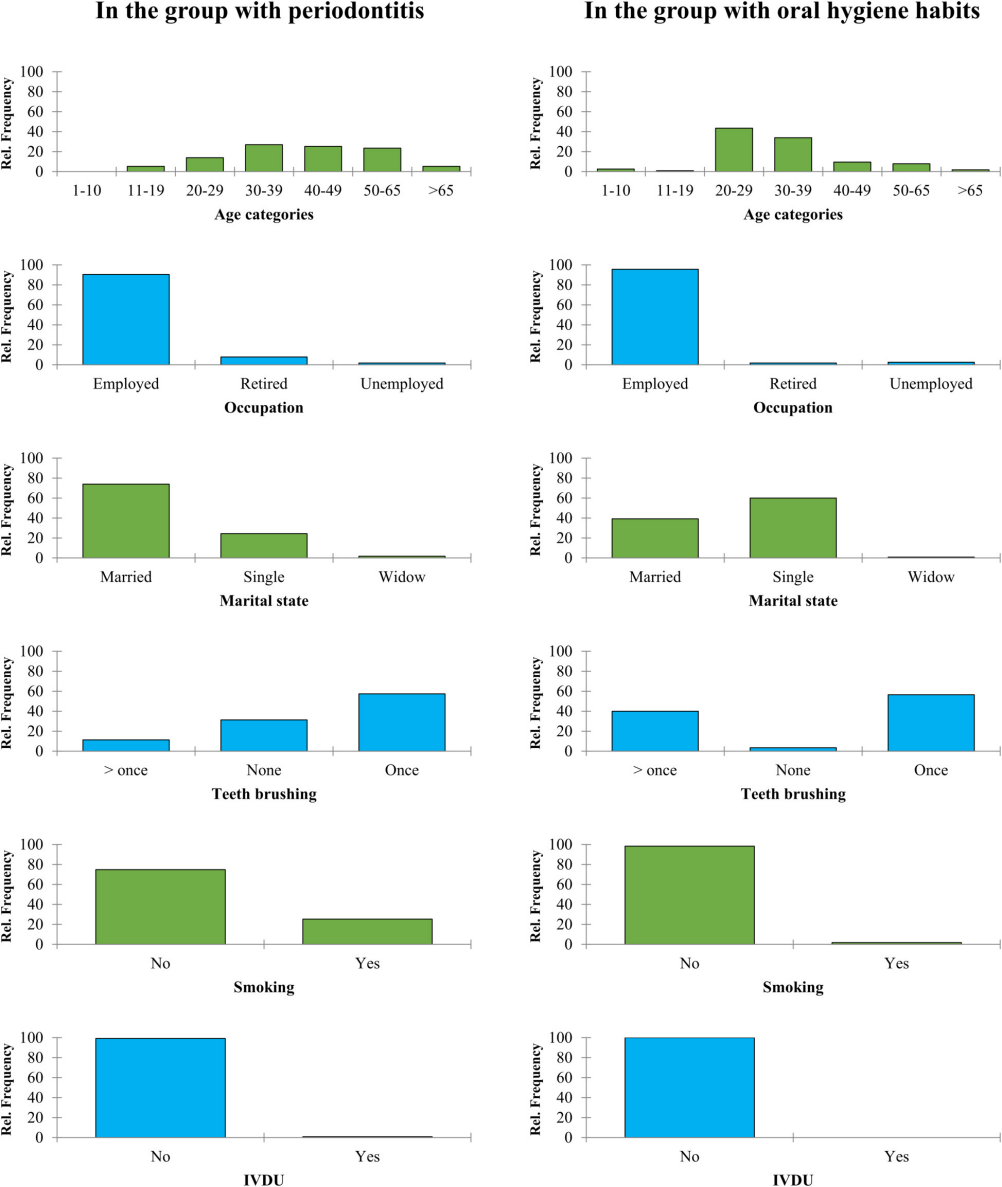
Demographical information retrieved from the participants in two case and control groups.

Further comparison of demographical findings between groups is depicted in Figure 1. In terms of age groups, most of the individuals in the periodontitis group were between the ages of 30–39 (26.96%) and 40–49 (25.22%), whereas the majority of the healthy group fell within the 20–29 (43.48%) and 30–39 (33.91%) age categories. The data on teeth brushing habits revealed that 57.39% of the periodontitis group brushed their teeth once a day, while 31.30% did not brush at all. On the other hand, 56.52% of the healthy group brushed once daily, with only 3.48% not brushing at all. In terms of smoking history, it was found that 74.78% of the periodontitis group did not smoke, while the healthy group had a higher percentage of non-smokers at 98.26% (see Figure 1).

### 3.2 The analysis of hygiene habits or health-related variables and medical history of underlying diseases

The participants reported underlying diseases such as anemia, asthma, hyperlipidemia, hypertension, diabetes, gastric surgery, Hashimoto's thyroiditis, cardiovascular disease, hyperthyroidism, hypothyroidism, rheumatism, thalassemia minor, and alopecia totalis (Table 2). Accordingly, 33/230 (14.35%) of participants reported underlying diseases. Among the participants in the periodontitis group, 24.35% reported having an underlying disease. In contrast, the healthy group reported a lower prevalence of underlying disease (4.35%).

**Table 2:** Data regarding the reported underlying diseases or complications by the participants (*n* = 230).

Variable\statistic	Nbr. of observations	Categories	Frequency per category	Rel. frequency per category (%)
Anemia	230	No	226	98.26
		Yes	4	1.74
Asthma	230	No	229	99.57
		Yes	1	0.43
Hyperlipidemia	230	No	228	99.13
		Yes	2	0.87
Hypertension	230	No	225	97.83
		Yes	5	2.17
Diabetes	230	No	224	97.39
		Yes	6	2.61
Gastric surgery	230	No	228	99.13
		Yes	2	0.87
Hashimoto's thyroiditis	230	No	229	99.57
		Yes	1	0.43
Cardiovascular disease	230	No	226	98.26
		Yes	4	1.74
Hyperthyroidism	230	No	225	97.83
		Yes	5	2.17
Hypothyroidism	230	No	222	96.52
		Yes	8	3.48
Rheumatism	230	No	228	99.13
		Yes	2	0.87
Thalassemia minor	230	No	229	99.57
		Yes	1	0.43
Alopecia totalis	230	No	229	99.57
		Yes	1	0.43

The prevalence of the underlying illnesses is presented in Table 3 in both the periodontitis and the healthy groups. In this regard, anemia was found in 3.48% of individuals in the periodontitis group, whereas the healthy group did not report it. 0.87% of the individuals in the periodontitis group had asthma, whereas none of the healthy group members reported having asthma. Hyperlipidemia was reported in 1.74% of individuals in the periodontitis group, while it was not present in the healthy group. 4.35% of the periodontitis group had hypertension, while none of the healthy group had it. 5.22% of individuals in the periodontitis group were found to have diabetes, while none of the individuals in the healthy group were reported to have the condition. A history of gastric surgery was reported by 1.74% of the periodontitis group, whereas the healthy group reported none. Hashimoto's thyroiditis was not seen in the periodontitis group, whereas it was reported by 0.87% of the healthy group. Cardiovascular disease had the same prevalence of 1.74% in both groups. 4.35% of the case group had hyperthyroidism, while none of the healthy group had the illness. 6.09% of the periodontitis group had hypothyroidism, compared to just 0.87% of the healthy group who had the same condition. Both rheumatism and thalassemia minor were found in 0.87% of the individuals in the periodontitis group, while none were present in the healthy group. No reports of alopecia totalis were reported in the periodontitis group, while it was found in 0.87% of the healthy group.

**Table 3:** Prevalence of underlying diseases among the studied groups with positive or negative PCR findings of reDoVs' genome.

Variable	State	Nbr. of observations	ReDoV	Frequency per category	Rel. frequency per category (%)
Anemia	Healthy	115	No	115	100.00
			Yes	0	0.00
	Periodontitis	115	No	111	96.52
			Yes	4	3.48
Asthma	Healthy	115	No	115	100.00
			Yes	0	0.00
	Periodontitis	115	No	114	99.13
			Yes	1	0.87
Hyperlipidemia	Healthy	115	No	115	100.00
			Yes	0	0.00
	Periodontitis	115	No	113	98.26
			Yes	2	1.74
Hypertension	Healthy	115	No	115	100.00
			Yes	0	0.00
	Periodontitis	115	No	110	95.65
			Yes	5	4.35
Diabetes	Healthy	115	No	115	100.00
			Yes	0	0.00
	Periodontitis	115	No	109	94.78
			Yes	6	5.22
Gastric surgery	Healthy	115	No	115	100.00
			Yes	0	0.00
	Periodontitis	115	No	113	98.26
			Yes	2	1.74
Hashimoto's thyroiditis	Healthy	115	No	114	99.13
			Yes	1	0.87
	Periodontitis	115	No	115	100.00
			Yes	0	0.00
Cardiovascular disease	Healthy	115	No	113	98.26
			Yes	2	1.74
	Periodontitis	115	No	113	98.26
			Yes	2	1.74
Hyperthyroidism	Healthy	115	No	115	100.00
			Yes	0	0.00
	Periodontitis	115	No	110	95.65
			Yes	5	4.35
Hypothyroidism	Healthy	115	No	114	99.13
			Yes	1	0.87
	Periodontitis	115	No	108	93.91
			Yes	7	6.09
Rheumatism	Healthy	115	No	114	99.13
			Yes	1	0.87
	Periodontitis	115	No	114	99.13
			Yes	1	0.87
Thalassemia minor	Healthy	115	No	115	100.00
			Yes	0	0.00
	Periodontitis	115	No	114	99.13
			Yes	1	0.87
Alopecia totalis	Healthy	115	No	114	99.13
			Yes	1	0.87
	Periodontitis	115	No	115	100.00
			Yes	0	0.00

### 3.3 ReDoVs prevalence in the participants

The presence of ReDoV's genome was investigated in 230 participants. The results showed that 63.48% (146 participants) tested negative for ReDoV, while 36.52% (84 participants) tested positive. Furthermore, comparing ReDoVs status between periodontitis and healthy groups revealed significant differences. Among the periodontitis group, 59 (51.30%) were ReDoV-positive. In contrast, 25 (21.74%) participants in the healthy group had ReDoV-positive results. The test of independence between groups and ReDoV status yielded a significant difference between the groups [*χ*^2^ value of 21.68 > the critical value of 3.84, df(1), *p*-value < 0.0001]. This indicates a significant association between periodontitis and ReDoV genome presence. Fisher's exact test confirmed the significance, showing that the number of ReDoV-negative cases in the healthy group and ReDoV-positive cases in the periodontitis group were significant. The cell-specific *χ*^2^ values indicated that the observed associations were considerable, further supporting the conclusion of an important link between group classification (periodontitis/healthy) and ReDoV status.

The odds ratio (OR) was calculated as 3.793, with a 95% confidence interval (CI) ranging from 2.136 to 6.727, indicating that individuals with periodontitis are approximately 3.8 times more likely to test positive for ReDoV than healthy individuals. The Z statistic of 4.66 and a highly significant *p*-value (<0.0001) lead to the rejection of the null hypothesis (H0: *ψ* = 1), suggesting a statistically significant difference in the odds of testing positive for ReDoV between the two groups.

### 3.4 The association of ReDoV status and demographic and behavioral oral hygiene factors

The association between ReDoV status and demographic and behavioral oral hygiene variables was investigated using *χ*^2^ and Fisher's exact test. The contingency table for sex and ReDoV status showed no significant association (*χ*^2^ = 1.97, *p-*value = 0.16). In contrast, age category and ReDoV status were significantly associated (*χ*^2^ = 20.02, *p-*value < 0.0001), suggesting a significant association between age and ReDoV prevalence. No significant association was found for occupation with ReDoV status (*χ*^2^ = 2.16, *p-*value = 0.34). Marital status and ReDoV status also showed no significant association (*χ*^2^ = 5.58, *p-*value = 0.06). However, teeth brushing habits were significantly associated with ReDoV status (*χ*^2^ = 19.78, *p-*value < 0.0001). In addition, a significant association between dental floss usage and the presence of ReDoVs among participants (*χ*^2^ = 9.29, df = 2, *p* = 0.0096). Smoking history was significantly associated with ReDoV status (*χ*^2^ = 5.19, *p-*value = 0.02), where smokers were more likely to be ReDoV-positive (odds ratio = 2.39, 95% CI: 1.12–5.09). Lastly, no significant association was found between intravenous drug use (IVDU) history and ReDoV status (*χ*^2^ = 1.75, *p-*value = 0.19).

**Figure 2:**
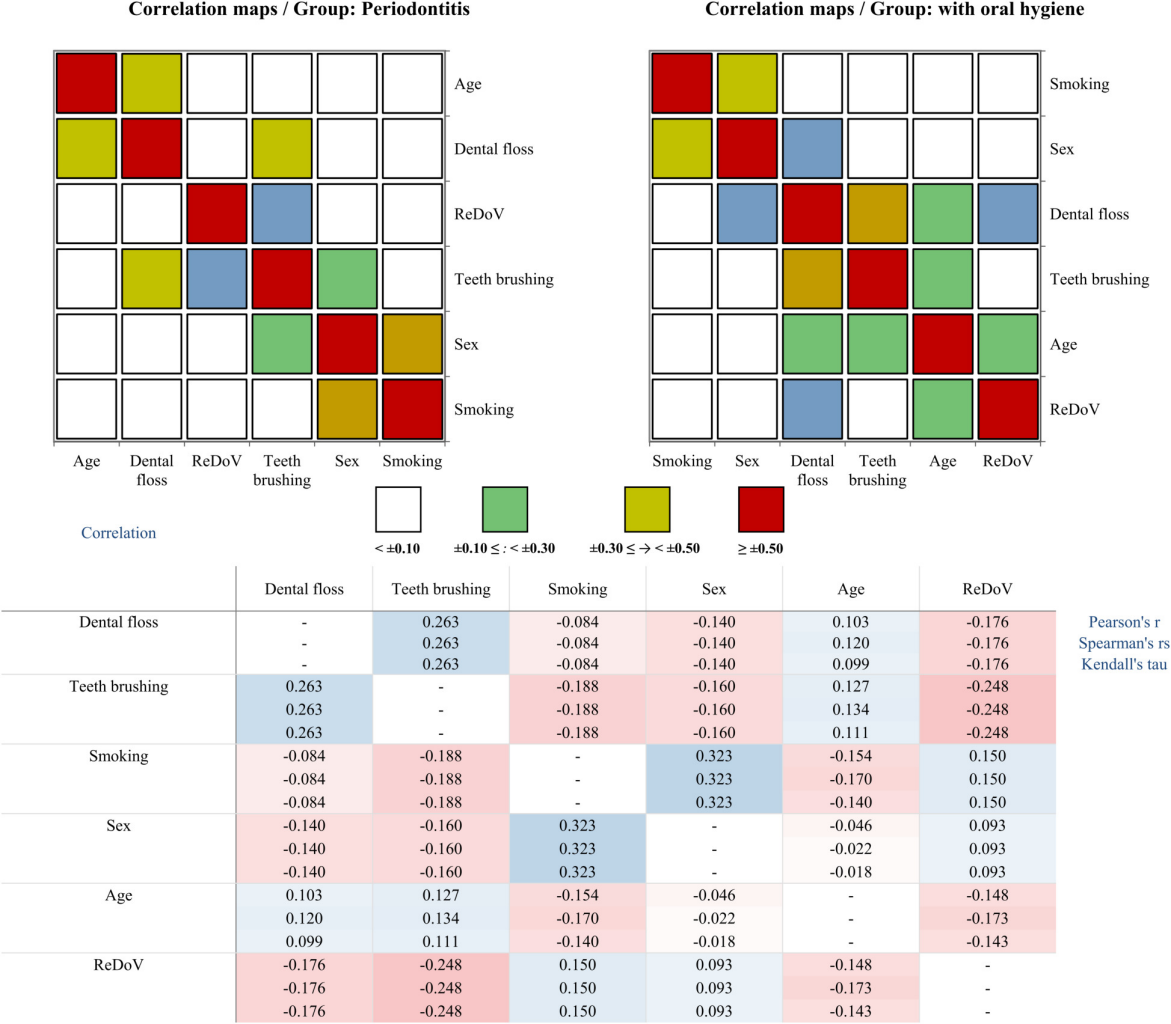
Correlation structure of ReDoV's genome presence and demographical and oral hygiene variables in two groups. Top panels: Pearson correlation “heat-maps” for the periodontitis group (left) and the oral-hygiene group (right). Each square represents the pairwise Pearson's r between two variables. Colors encode the magnitude of the correlation: white for negligible, green for weak, yellow for moderate, and red for strong. Axes are labeled with each variable and the diagonal cells are omitted for clarity. Bottom panel: Numerical correlation matrices showing three measures, including Pearson's *r*, Spearman's *ρ*, and Kendall's *τ* of association for each variable pair. Cells are shaded on a red–blue divergent scale. Dashes (–) indicate self-correlations.

As shown in Figure 2, Pearson correlations in the prople with oral hygene habits revealed that better flossing habits were strongly linked to more frequent tooth brushing (*r* = 0.25, *p* = 0.01), and, conversely, flossing was associated with reduced detection of ReDoV genomes (*r* = –0.20, *p* = 0.03). Accordingly, participants who reported regular floss use had 31.3% lower odds of ReDoV detection compared to those who did not floss (OR = 0.687, 95% CI: 0.521–0.907). A likelihood-ratio test confirmed that this model fit the data significantly better than the null model (*Δ*G^2^ = 7.21, df = 1, *p* = 0.0073). No other associations with smoking, age, or brushing frequency reached statistical significance in this group. By contrast, among participants with periodontitis, the only oral-hygiene behavior tied to viral presence was tooth brushing, which showed a small but significant inverse relationship with ReDoV detection (*r* = –0.21, *p* = 0.03). In this subgroup, smoking and sex were also significantly correlated (*r* = 0.40, *p* < 0.0001), but neither smoking nor age related significantly to viral status.

### 3.5 The association between ReDoV status and underlying diseases

No significant association was found between the presence or absence of the ReDoVs genome and underlying diseases reported by the participants. Further analysis included a contingency table comparing individuals with and without diabetes against their ReDoV status was performed. It was observed that among the 230 individuals, 145 individuals without diabetes tested negative for ReDoV, while 79 individuals without diabetes tested positive for ReDoV. In contrast, only 1 individual with diabetes tested negative for ReDoV, whereas 5 individuals with diabetes tested positive for ReDoV [*χ*^2^ value of 5.82 > critical value of 3.84, df(1), *p-value* = 0.02]. Fisher's exact test further confirmed this significance, with diabetic participants being more likely to be ReDoV-positive. In support of this finding, the calculated odds ratio was 9.18 (95% CI: 1.48–56.98), although this was based on a very small diabetic subgroup (*n* = 6).

### 3.6 Phylogenic analysis

**Figure 3:**
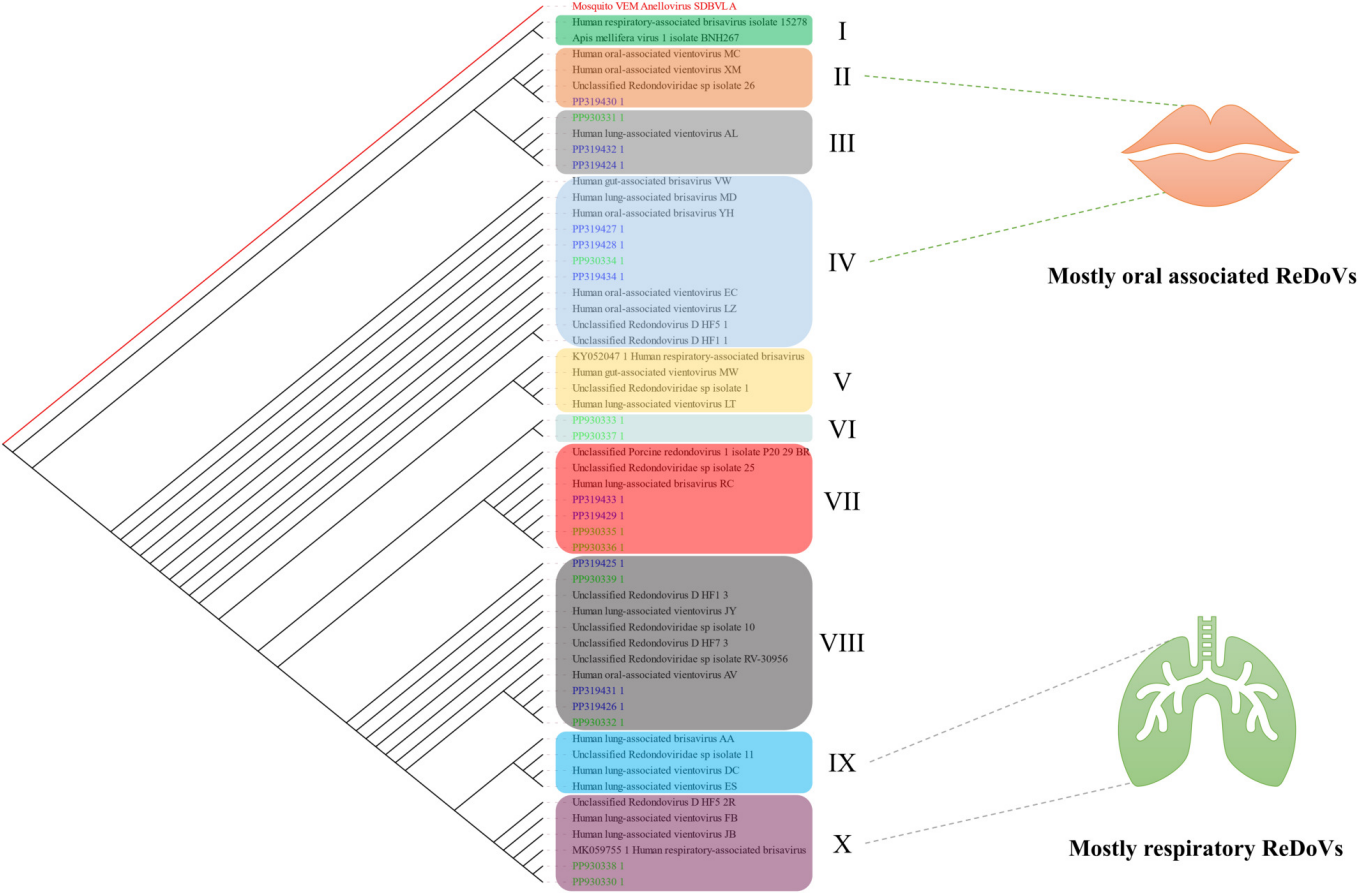
A slanted cladogram showing the ReDoVs' genomic data based on their genetic distance. Data obtained from the present study are depicted in green, and those from the previous study are in blue. Different clades were established based on the formed association of leaves (ReDoVs). The tree is re-rooted with Mosquito VEM Anellovirus SDBVL A (NC_076121.1) according to our previous study (14). The unidentified ReDoVs were ignored for clustering clades II and IV or IX and X since they could already belong to these categories.

Randomly, ten samples were sequenced and submitted to GenBank with the accession numbers PP930330 to PP930339. Furthermore, data from the previous study was also retrieved with the accession numbers PP319424 to PP319438. As illustrated in Figure 3, several clades (I–X) could be established, with few with consistent organ associations. Accordingly, clades II and IV comprised the genomic data of ReDoVs with mostly oral cavity distribution. Also, clades XI and X comprised respiratory- (lung) associated ReDoVs. Other clades represented a mixture of different organ-associated or unidentified ReDoV genomes.

## 4 Discussion

The primary study by Abbas et al. (6) reported a high prevalence of ReDoVs in periodontal tissues and suggested that these viruses might play a role in the pathogenesis of periodontitis (6). This study was also considered to confirm the findings of our previous study (14). In the preceding research, due to the type of study, we could only manage to interview the referrals for any dental or gum issues. During the interview, the participants acknowledged their gum or dental complications; whether their problems were due to periodontitis was unclear. In the study, however, it was found that individuals with periodontitis had a higher presence of the ReDoVs genome (51.30%) than those with oral hygiene habits (21.74%). This is also aligned with a report that identified a correlation between ReDoV and chronic periodontitis (23). We further noticed poor oral-hygiene markers, especially reduced flossing, were significantly associated with ReDoV detection. This indicates that oral hygiene practices might participate in the occurance of ReDoVs. Therefore, in the present study it was aimed to evaluate if ReDoVs would be more prevalent in individuals with chronic periodontitis, especially those with poor oral hygiene.

Recent studies have revealed a complicated interplay between viruses and periodontal disease. Abbas et al. (6) discovered the *Redondoviridae* family and reported that redondovirus levels are elevated in subjects with periodontitis, decreasing after treatment (6). Zhang et al. (23) later found a significantly higher prevalence of ReDoVs in periodontitis patients (72%) than in healthy controls (52%) (23) identifying several novel strains. More recently, it was demonstrated that ReDoVs likely replicate within the oral protozoan *E. gingivalis*, highlighting an indirect association with periodontal disease (24). These studies, alongside emerging data on the oral virome (14), suggest that viruses like ReDoVs could be an integral part of the periodontal microbial ecosystem. In this regard, demographic and behavioral factors, such as age, oral hygiene practices, and smoking history, were significant determinants of ReDoV prevalence in both (23) and this study. The significant age disparity between groups (mean age 41.05 vs. 33.17 years) may confound results, as aging is linked to immune senescence and increased susceptibility to infections.

Here, it was found that dental floss is significantly associated with a lower incidence of periodontitis and less frequency of the ReDoV genome in those who frequently use dental floss. This further supports the complex multifactorial impact of ReDoV on dental health. Accordingly, ReDoVs may exploit *Entamoeba gingivalis* (*E. gingivalis*) as a host in the oral cavity, potentially influencing the pathogenicity of periodontal disease (24, 25). Therefore, we suggest a possibly higher incidence rate of ReDoVs in those with poor oral hygiene and less dental floss use. However, overall poor health, which is not addressed in the present study, can confound the observed association between ReDoV detection and periodontal status because it is linked both to the periodontitis or poor oral hygiene and to the higher viral prevalence. This is supported by the studies reporting individuals in poorer general health are more prone to chronic inflammatory conditions, including periodontitis (26, 27).

In our study we defined chronic periodontitis according to the 2018 AAP criteria characterized by gradual attachment loss and pocket formation in the presence of dental plaque, Aetna. We did not assess other recognized forms, such as the rapidly progressing “aggressive periodontitis” or the necrotizing variants. Future work should therefore determine whether ReDoV prevalence and its inflammatory impacts differ across these distinct clinical phenotypes of periodontal disease. Periodontitis is identified as an inflammatory condition affecting the periodontium, causing tissue damage and loss of alveolar bone (28). It is common among adults, leading to a considerable impact on public health (29). Periodontitis is also associated with local immune system alteration and systemic diseases (30, 31). This supports the establishment of viral infection in the gastrointestinal and respiratory tract axes. As a result, the occurrence of underlying diseases was higher among participants in the periodontitis group (24.35%) than in those with oral hygiene habits (4.35%). Significantly, a relationship was identified between diabetes and ReDoV status. Nevertheless, only 6 individuals in this study had diabetes, all belonging to the periodontitis group, highlighting the necessity for further investigation.

Approximately half of the periodontitis patients harbored ReDoVs. This association in prevalence was notable, but its biological significance is not yet clear. Also, the findings of the present study do not establish causation, and it remains unclear whether ReDoVs actively contribute to periodontal tissue destruction or are merely incidental bystanders as ReDoVs often coexist with the oral protozoan *E. gingivalis*, which is enriched in periodontitis lesions (24). To date, no direct pathogenic mechanism for ReDoVs in periodontal disease has been confirmed, and ReDoVs are not known to cause disease in humans despite their association with illness. However, the etiologic role for ReDoVs cannot be ruled out, an alternative explanation is that poor oral hygiene and chronic inflammation allow opportunistic colonization by organisms that harbor ReDoVs*.* Nevertheless, the findings of this study emphasize that the higher viral detection rate in disease was mainly due to poor oral hygiene. Particularly, flossing can be a good predictor of ReDoV infection.

The ReDoVs have been identified in different tissues. To further classify and to investigate the genetic distance between the isolates of ReDoVs, a phylogenetic analysis was performed. In this regard, a highly sensitive and accurate progressive multiple sequence alignment (MSA) was done using data provided by the NCBI's Taxonomy database and our sequencing results. To cluster the ReDoVs as a group, the tree was rooted with a closely related member of Annelovirus. There are currently a few categories, including the human gut, lung, oral, respiratory-associated Brisaviruses or Vientovirues, alongside unclassified and non-human ReDoVs. These groups lack enough members to accurately and robustly distinguish them. Also, it is poorly understood that lung-associated ReDoVs could be associated with oral or respiratory ReDoVs and vice versa. Additionally, the detected isolates phylogenically clustered among oral/respiratory-associated ReDoVs described in prior research, rather than forming completely distinct lineages. However, this analysis was based on partial genome sequencing, and we did not assess functional properties of the viruses. At present, there is no evidence that the ReDoV strains in oral cavities differ in behavior or pathogenicity from strains found in other sites; our data only indicate genetic relatedness.

The results of the present study demonstrated a high prevalence of the ReDoVs genome in adult individuals with periodontitis who are less concerned about dental hygiene. Future research should focus on further oral hygiene and overall poor health factors to identify indicator(s) of ReDoV infections.

## 5 Conclusion

This study demonstrates a significant association between ReDoVs and periodontitis, particularly in individuals with poor oral hygiene. Poor oral hygiene and periodontal disease are a good indicators of ReDoV infections. In other words, people with poor oral hygiene are more likely to test positive for the presence of ReDoVs genome. Particularly, flossing was negatively correlated with the ReDoVs presence, and the prevalence of infection was higher in those with poor oral hygiene. Consequently, the increased occurrence of the virus in patients with periodontitis may indicate the effects of periodontal issues and inadequate oral hygiene instead of being a caus. Phylogenetic analysis identified distinct clades with a mixture of ReDoVs with different origins.

## Data Availability

The datasets presented in this study can be found in online repositories. The names of the repository/repositories and accession number(s) can be found in the article/Supplementary Material.
