# Supplementary material for: Redondovirius-associated periodontitis in people with poor oral hygiene: a cross-sectional study
Source: Front Oral Health. 2025 May 23;6:1572274. doi: 10.3389/froh.2025.1572274 (PMC12142335; doi:10.3389/froh.2025.1572274)
Supplement: Supplementary file 1 [file Datasheet1.pdf]

Questionnaire of a proposal entitled “Investigation of the presence of Redondovirus infection in gingival samples of patients with moderate to severe periodontitis and healthy individuals visiting health centers affiliated with Iran University of Medical Sciences“

Project Code: 85162

Ethical code of the study: IR.IUMS.REC.1402.530

Note 1: The implementation of this study is subject to ethical considerations.

Individual code: Date of Sampling:

General

1. Gender

Male ☐ Female ☐

2. Age: …

3. Occupation

Employed ☐ Retired ☐ Unemployed ☐

4. Marital status

Single ☐ Married ☐ Divorced ☐ Widowed ☐

5. Reason for visit (verified by the dentist)

Healthy gums ☐
Unhealthy gums ☐
Specification ……………………………………………………………………………………..

Medical history

1. Does the patient have any underlying diseases? (If yes, please specify)

Yes ☐ No ☐
Specification: ………………………………………………………………………………

2. Has the patient had respiratory symptoms during the visit? (If yes, please specify the symptoms)
Yes ☐ No ☐
Specification ……………………………………………………………………………….

3. Has the patient ever had a test for respiratory viral infections like COVID-19?
Yes ☐ No ☐
If yes, what were the results? Positive ☐ Negative ☐

4. Does the patient have a history of the SARS-CoV-2 or any other respiratory infection? (If yes, please specify the infection)
Yes ☐ No ☐

Habits

1. Smoking history
Yes ☐ No ☐

2. History of addiction or drug abuse
Yes ☐ No ☐

3. How often does the patient brush their teeth?
Doesn't brush ☐ Once a day ☐ More than once a day ☐

4. Does the patient use dental floss?
Yes ☐ No ☐

If you have any questions or concerns about this study, please get in touch with the investigator(s) at the following contact addresses:

+98 935467 4593

Alirezaa2s@gmail.com
